# Patients' and healthcare professionals' experiences with implementing the Rosa chatbot in mainstream genetic testing for hereditary breast and ovarian cancer

**DOI:** 10.1002/jgc4.70119

**Published:** 2025-10-23

**Authors:** Elen Siglen, Hildegunn Høberg Vetti, Anita Lyssand, Tone Dahl‐Michelsen, Cathrine Bjorvatn

**Affiliations:** ^1^ Faculty of Health Studies VID Specialized University Bergen Norway; ^2^ Western Norway Familial Cancer Center, Department of Medical Genetics Haukeland University Hospital Bergen Norway

**Keywords:** BRCA1, BRCA2, breast cancer, chatbot, digital tool, digitizing healthcare, genetic counseling, HBOC, implementation barrier, mainstream genetic testing, ovarian cancer, patient‐centered care

## Abstract

Mainstream genetic testing (MGT) refers to genetic testing conducted at the time of a cancer diagnosis without undergoing comprehensive genetic counseling. MGT has been the standard of care for patients with breast or ovarian cancer in Norway for several years. The aim of this study is to explore how newly diagnosed patients with breast or ovarian cancer and healthcare professionals' (HCPs), experience the use of the Rosa chatbot in mainstream genetic testing (MGT) and explore potential barriers to the implementation of chatbots in MGT. We conducted a qualitative study using semi‐structured interview guides with selected patients and HCPs. The interviews were done either: in‐person, over the digital platform Teams, or over the telephone, depending on the participants' wishes. We chose the Stepwise‐Deductive Inductive approach for analyzing the transcripts. Both patients and HCPs viewed the Rosa chatbot positively, describing it as user‐friendly, useful, accessible, safe, professional, and trustworthy. They reported that the volume and complexity of information during MGT could be overwhelming and viewed the chatbot as a trustworthy resource for patients to revisit at their own pace, supporting informed decision‐making after a positive genetic test result. However, concerns were raised about potential misunderstandings, the impersonal nature of digital communication, and the risk of reduced patient–provider interaction, which together were perceived as an emotional barrier to integrating chatbots into genetic counseling practice.

**What is known about this topic**

Content should be presented in a tailored and relevant manner suitable for the  patients' individual situation, with the aim to be perceived as useful, user‐friendly, and trustworthy when implementing digital health technologies. As cancer patients may be generally amenable to incorporating digital health technologies into their care, it has been suggested that future research should be directed towards exploring strategies for successful implementation of digital health technologies into routine cancer care practice. This should include identifying both individual and institutional‐level barriers and facilitators to the use of such technologies.

**What this paper adds to the topic**

This study sheds new light on how we should prepare for a future where hybrid health services are inevitable and digital information services will play a significant role. Both patients and HCPs have positive attitudes towards the Rosa chatbot as a trusted supplement to genetic testing; however, they highlight the importance of human interaction to validate the information provided by the digital tool. Subsequent to our interviews with both cancer patients and HCPs in a mainstream setting, we have identified a "fear of missing out on human interaction" as an emotional barrier to the implementation of a chatbot in these groups. Through a patient‐centered care framework, it can be assured that the chatbot serves as a supplement only, allowing for face‐to‐face human contact as needed.

## 1 INTRODUCTION

Traditionally, the heart of genetic counseling has traditionally been the therapeutic relationship between a counselor and patient where the focus has been on providing information, facilitating decision‐making, and fostering adaptation when a patient is faced with a new reality of having a hereditary disease or risk (Biesecker, 2020). In recent years, digital technology has been developed and applied to every aspect of health and healthcare (Abernethy et al., 2022), including genetic services (Gordon et al., 2018; Kohut et al., 2019). In such digitalized healthcare services, tools easy to validate, curate, and share hold the promise of easing provider burden and augmenting clinical reasoning skills (Abernethy et al., 2022). Matching the service delivery model to the patients' needs is essential to achieve effective outcomes. The key challenges are identifying which elements are (not) suitable for digitizing and, for which patients these tools may or may not be suitable for, ensuring that those with the greatest need of in‐person counseling have access to a genetic counselor (Biesecker, 2020).

Mainstream genetic testing (MGT) refers to genetic testing conducted at the time of a diagnosis, in this study being a breast or ovarian cancer diagnosis, without comprehensive genetic counseling (Bokkers et al., 2022; Rahman, 2014). MGT of patients with breast or ovarian cancer has been standard care for several years (Bokkers et al., 2022; Høberg‐Vetti et al., 2016), and includes testing of the *BRCA1* and *BRCA2* genes, with various selections of additional genes (Rahman, 2014) depending on local or national guidelines. In the setting of mainstream testing in Norway, pre‐test information typically consists of a brief explanation provided by the ordering physician accompanied by an information sheet. If a pathogenic variant is detected, the patient will be offered, and referred to, genetic counseling with one of the medical genetics departments in the country. International studies have found that patients with breast cancer prefer the mainstream pathway (Catherine Beard et al., 2024); however, it is important to provide genetic counseling for those receiving a positive test result (Al‐Hilli et al., 2023; Beard et al., 2024).

In this context, we wanted to implement the Rosa chatbot as a digital information tool focused on hereditary breast and ovarian cancer. The Rosa chatbot was developed and evaluated between the years 2018 and 2022 (Siglen et al., 2022, 2023) and contains extensive information about genetic testing with a focus on information about the BRCA genes, surveillance programs, family communication, and more. Using the chatbot allows the patients to personalize the information flow and read and repeat as they wish. Rosa is a chatbot designed to provide reliable information about hereditary breast and ovarian cancer. Built on a commercially available platform with Norwegian language support, it uses machine learning and natural language processing to understand patient questions and retrieve predefined answers. These responses, developed by genetic counselors and geneticists from all health regions in Norway, ensure accurate and trustworthy information. Rosa does not generate its own answers. Patients can access Rosa through a mobile app, where they can interact via chat, explore additional resources through “read more” buttons, watch educational videos, and find links to relevant websites.

In their review of seven studies examining the use of chatbots for genetic cancer risk assessment and pre‐test counseling for cancer genetic testing, Webster et al. (2023) reported that chatbots have the potential to reduce the time burden and complexity associated with genetic cancer risk assessment for clinicians. They further noted that the interactive nature of chatbots, coupled with their ability to deliver personalized educational content, may enhance patient engagement and understanding of genetic testing. Consequently, chatbots appear to hold promise for improving patient care and outcomes. Although chatbots have a strong potential to increase access to genetic services for unaffected individuals (Kaphingst et al., 2024), it can be questioned if this applies also to the mainstream setting. Rosa has previously been evaluated by healthy individuals at risk of hereditary breast and ovarian cancer. Relatives in families with suspected or confirmed hereditary breast and ovarian cancer valued Rosa as a trustworthy and easily available support tool (Siglen et al., 2023). Rosa has not been evaluated by cancer patients before this study. As genetic testing is performed in the early diagnostic setting, we selected this context for the final iteration of the Rosa app in the Rosa chatbot project. The aim of this study is to explore how newly diagnosed patients with breast or ovarian cancer and healthcare professionals' (HCPs) experience the use of the Rosa chatbot in mainstream genetic testing (MGT), and explore potential barriers to the implementation of chatbots in MGT.

## 2 METHODS

### 2.1 Study Design

Between December 1st, 2023 and May 31st, 2024, 335 newly diagnosed breast and/or ovarian cancer patients in the Western Norway health region were invited to use the Rosa chatbot for information about the genetic test alongside MGT. Clinicians introduced the Rosa chatbot to patients during consultations when discussing genetic testing. Alongside standard written and oral information, the chatbot was presented as an additional resource. All patients offered genetic testing also received a flyer with details about the chatbot and how to access it. Clinical nursing staff reinforced this in follow‐up consultations, checking whether patients were aware of the chatbot, needed assistance with downloading it, or had any questions.

A qualitative interview study was conducted with selected patients and HCPs using the chatbot in the given time frame. These patients were selected based on age and diagnosis to ensure a diverse sample. A total of 10 patients were contacted; one did not respond, while the remaining nine all consented to participate in an interview. Two interviews were discarded from further analysis as the participants had not used the chatbot, leaving us with six interviewed patients with breast cancer and one with ovarian cancer. Both excluded participants were over 70 years old. HCPs in our health region, responsible for organizing MGT for patients with breast or ovarian cancer, were invited through ward managers. We targeted a diverse sample from both breast and ovarian cancer clinics, nurses, gynecologists and oncologists being represented. A total of 11 healthcare professionals (HCPs) were eligible, but due to one no‐show, 10 interviews were conducted.

The interviews were held either in‐person (4 patients and 5 HCPs), over the digital platform Teams (0 patients and 5 HCPs), or by telephone (3 patients and 0 HCPs), depending on the participants' preferences. The interviews were conducted and transcribed by the first author (ES). The transcripts were made available to all authors, with audio files available to two of the co‐authors (CB and HHV). The transcripts were coded in NVivo (QRS International) by the first author.

We developed two separate semi‐structured interview guides, one for the patient interviews and one for HCPs. The patient interview guide focused on the following questions (with probes): (1) What are your experiences with genetic testing alongside receiving a cancer diagnosis? (2) How did you feel about using the Rosa chatbot during MGT? (3) How did you perceive the information and communication with the hospital during this early stage? (4) What are your experiences with digitalization, and how has it influenced your relationship with health services? The HCP interviews concentrated on the following questions (with probes): (1) How did you introduce the Rosa app to your patients? (2) What potential do you see in this type of communication tool? (3) How do you think digital tools like Rosa will impact health services? (4) What are your thoughts on implementing the Rosa chatbot in clinical care? After conducting the interviews, we reviewed the interview guide related to both groups, resulting in some added probes regarding experiences with digitalization.

### 2.2 Data Analysis

We chose the Stepwise‐Deductive Inductive approach for the analysis of the interview transcripts, as described in Tjora (2018). This model outlines a research process where thorough data analysis is key to concept development. The model operates on an inductive principle, starting with raw data and progressively moving towards concepts or theories through six steps via incremental deductive feedback loops, thus named the “Stepwise‐Deductive Induction” (SDI) approach. In the analysis phase, it is emphasized that the researcher(s) keeps close contact to the empirical data when shaping the codes.

The first three steps focus on the coding process, ensuring relevant empirical codes. Once all the transcripts are coded, the fourth step is to group codes with similar content. The code groups are deductively tested by checking that the codes in one group are thematically different from the other groups and, at the same time, are internally consistent. In the fifth step, one goes from an inductive approach to an abductive approach. In this step, the researcher moves away from the empirical data, interprets the groups, and tries to theorize or make new concepts, seeking a more general label to describe the phenomenon. The deductive element in this step is the validation of the findings in relevant literature to question whether there are already known theoretical contributions shedding light on this subject.

The interviews were conducted in Norwegian, and the analytical process was carried out in Norwegian until the conceptual stage. We had three research meetings discussing themes and moving codes back and forth between groups. The transcripts remained untranslated throughout the analysis. All co‐authors except AL were present at all research meetings. When discussing concepts, the names assigned to each code group were translated to English. Thus, the concepts were developed exclusively in English.

## 3 RESULTS

### 3.1 Participants

The analysis is based on interviews with six patients with breast cancer and one patient with ovarian cancer, carried out 10–14 weeks after they underwent genetic testing. They ranged in age from 28 to 57 years. All were female and had attained at least a vocational education. Four had a spouse, five had children, and six had received their genetic test results before the interview. Five of these six had received a negative genetic result, and one had received a positive result. One patient had not received the result before the interview and was unaware of her mutation status. The interviews lasted 30–90 min.

We also interviewed 10 self‐selected HCPs about their experiences using the chatbot in the mainstream setting. Of the 10 HCPs, four were nurses (two oncology nurses and two nurses working in breast clinics) and six were physicians (two gynecologists, two breast surgeons, and two oncologists), with only one male participant. All worked in hospital units in Western Norway providing MGT to this patient group. The HCP participants ranged in age from 29 to 53 years, with experience spanning from 5 years to over 20 years. The interviews lasted 25–40 min.

### 3.2 Chatbot Performance

The specific chatbot use of individual participants was not recorded due to privacy regulations: only overall usage was approved for recording. Throughout the study period, there were 336 entries to the Rosa app. These provided a total of 1140 questions, with an average of 3.4 questions per entry (range 1–29). Of the 1140 answers given, we registered 152 (13.3%) fallback answers and 74 (7.5%) mismatched (wrong) answers. A fallback answer is chatbot terminology for a predefined response (e.g. “I am sorry, I don't understand (…)”) triggered when the chatbot cannot match the user's input to any existing rule or intent. A mismatched answer is when the chatbot gives a response that does not match the user's question, often because the system incorrectly interprets the input or applies the wrong rule. In contrast to how Rosa performed when evaluated by healthy individuals at risk of breast or ovarian cancer (Siglen et al., 2023), the mainstream population in this study used the app more broadly, and more questions fell outside the scope of the chatbot. They asked questions about prognosis, treatment, and surgery, which led to reduced accuracy (88.7% in the group of healthy individuals vs. 80.1% in the MGT group), increased levels of fallbacks (9.7% vs. 13.3%), as well as increased levels of mismatched answers (1.8% vs. 7.5%).

### 3.3 Arriving at Concepts

We generated 242 empirical codes based on the HCP interviews, and 199 based on the patient interviews. The code lists were reviewed and validated by three co‐authors,^1^ and all codes passed the deductive code test. The code lists were analyzed separately up to the fourth step of the SDI model. The codes were grouped, and this analytical phase resulted in nine thematic groups from the HCP interviews and 10 from the patient interviews. Some of the thematic groups fell outside the scope of the study aim and were thus excluded.

This left us with seven thematic groups based on the patient interviews, and eight based on the HCP interviews. Next, these thematic groups were grouped together and developed into concepts. Concept 1: “A trusted supplement to genetic testing” included the following thematic groups: opinions about Rosa, attitudes towards chatbots, and potential for chatbots. Concept 2: “A tool for balancing facts, fears and hope” included the following thematic groups: information seeking versus information avoidance, the importance of hope, and perceived patient concerns. Concept 3: “A valued support for decision‐making” included: thoughts about digital communication, reflections about mainstream genetic testing, and patient information needs. Finally, concept 4: “An impersonal tool – Fear of missing out on human interaction” included: the value of the physical meeting, fear of misunderstanding digital information, what is at stake when digitizing, and barriers to implementation.

### 3.4 Description of Concepts

#### 3.4.1 Concept 1: A trusted supplement to genetic testing

Both the patients and the HCPs considered the Rosa chatbot as a trusted supplement to genetic testing. Their trust here included a positive evaluation of the chatbot, positive attitudes about the chatbot, and the potential they see in Rosa as a supplementary information tool in the mainstream setting.

It helped me know a little more about what I might need to ask the doctor about and what I didn't need to ask about. (P4)

Words as user‐friendly, useful, available, safe, professional, and trustworthy were typically mentioned in the interviews in both groups. An illustration of how the chatbot served as a trusted supplement is provided by one patient saying:

I accessed Rosa and found the information I needed immediately (…) When I waited for the genetic test results, I wondered how long it would take before I would receive them and found that it could take six to eight weeks. I also wondered what would happen after. So, I used it for information. (P6)

Typically, they saw the Rosa chatbot as relevant and underscored it as objective and thus trustworthy. As hereditary cancer was an unfamiliar topic to them, the feeling of objectivity might boost the feeling of Rosa as trustworthy. One of the patients said:

It is objective. Fact‐based. Easy and quick to navigate. So, it has been useful. I have visited it afterward as well. (P6)

In general, all HCPs had a positive attitude towards using digital tools such as the Rosa chatbot in healthcare. They viewed it as a useful and trusted information tool supplementing the genetic testing process, particularly for providing consistent and accessible genetic information. Statements such as “It felt safe to use” and “It looked professional” were expressed by several of the HCPs.

This was cool and different. It's a good tool for our patients. (HCP1)

They see how this type of technology may relieve and improve healthcare services. They rate the Rosa chatbot over Google for the purpose of genetic information in the MGT setting and see it as a valuable and trusted support tool.

I believe the potential for this type of information channel is great. (HCP1)

They further see the possibility of standardizing the information for all through a chatbot.

It's the same for everyone, no matter who it is, they see the same information (…) It's almost like a checklist or a procedure, which I think is great. And when the information is written in a clear and understandable language, it makes it easier for patients to comprehend. (HCP6)

#### 3.4.2 Concept 2: A tool for balancing facts, fears and hope

The Rosa chatbot was described as a tool where the patients could select for themselves what information they wanted to read, avoid topics that felt intimidating, and in that way maintain hope. All the participants highlighted the importance of thorough information in the diagnostic setting. At the same time, they all said the amount of information at this stage is overwhelming to such an extent they had to take 1 day at a time, focusing on pulling through.

I couldn't take in so much information during the acute phase. (P2)

In that context, several mentioned the chatbot as a positive thing, as they could choose what to read and when; thus, they could also avoid topics they did not want to read about just yet.

I got more than enough information, at least after I downloaded the app (…) In Rosa, you can at least choose for yourself what you want to know and not know. I mean you can decide what you want to read and what you don't. (P5)

The balancing aspect of facts, fears, and hope was significant among the patients. They focused on the positive news and described that the ability to take in facts increased with the reception of positive news, as positive news provides hope of recovery.

The HCPs expressed wanting to help patients navigate the overwhelming flow of information, and valued the chatbot as a trusted resource they could confidently recommend for reliable information. They want to do what is best for the patients, and they acknowledge the patients' needs and concerns in this situation, knowing that patients receive more information than they can digest in the MGT setting.

They can't quite cope with it (the genetic test) when they've just received a cancer diagnosis. Their focus is directed towards the cancer treatment. (HCP7)

The HCPs highlight the importance of being gatekeepers of the truth, a place where the patients can clarify misunderstandings, underpinning the importance of a chatbot being a supplement, not a replacement.

There are questions and answers that are difficult to tailor in a chatbot (…) It can easily be a bit wrong, and that must be avoided. We must not provide misinformation. (HCP1)

#### 3.4.3 Concept 3: A valued support for decision‐making

The Rosa chatbot was considered a valued support for decision‐making and indeed as a support tool providing accurate information during the testing process.

I think all information is good to have. That way, you can make the choices you need based on good information. (P4)

All participants said they were happy to take the genetic test and felt it was a positive thing amidst it all. Comments like, “I'd rather know than put my head in the sand” were repeated by several, followed by an intention to help family members if a pathogenic variant had been detected. The Rosa chatbot was described as a useful tool for clarification.

We thought it could only come from the mother's side. So, I went in and asked about it and learned that the chance was just as high. It can come from both sides. So, I found it (the Rosa chatbot) very useful. (P4)

Two participants even said that they believed the detection of a pathogenic variant would have brought the family closer together as it would be something they would navigate collectively (P3 and P4). P6 illustrated how the chatbot facilitated decision‐making for her:

I went in and looked at the Rosa chat quite a bit when I got it. And I thought, well, if I have it, then it's about the breast and ovaries. And in that case, I can at least remove them. (P6)

They expressed that they highly appreciated being offered the genetic test and viewed Rosa as a positive asset in a  diagnostic setting, providing them with the information they needed to make future decisions. When asked if they felt the need for more thorough *pre‐test* counseling, all seven declined. However, they all said they would have needed genetic counseling if the test had been positive. A digital tool alone would not suffice if a pathogenic variant had been detected. This was consistent through all patient interviews that participants would like to speak with a human if a mutation was to be detected. Many also valued the chatbot in that context, saying they would have used it more if a mutation had been detected.

HCPs value the chatbot's curated content and that patients can rely on it as a trustworthy reference tool when making health‐related decisions.

The chatbot provides neutral information, not influenced by what I think is important information, which may make it easier for patients to make their own decisions. (HCP6)

They further see great potential in Rosa.

I definitely think that digital solutions from the healthcare system, that are evidence‐based and make it easier to find the right information, are a good thing (…) I liked the Rosa chatbot. I definitely think it is a useful tool for our patients. (HCP3)

#### 3.4.4 Concept 4: An impersonal tool –fear of missing out on human interaction

The use of a digital tool like Rosa in a sensitive setting can create a fear of missing out on essential information and human interaction. While Rosa provides valuable information, many felt it lacks the depth, empathy, and personalized support that human interaction offers, and in this sense, they see Rosa as an impersonal tool. One thing the patients fear is the risk of misunderstandings:

I'm afraid of missing the chance to double‐check that I understand correctly (…) I want to talk to a doctor. Because when you read something, you will always have questions. (P4)

The time they get to spend with a physician is highly valued. They instantly trust the information provided by a physician and feel seen and validated as a person. With access to digital information tools, the doctor may expect them to come prepared and may leave out information that would otherwise have been included in the consultation.

I'm afraid of losing important information, of course. (P7)

This feeling of responsibility to read and learn on your own time is mentioned by some. Although a digital tool may provide highly accurate information, even endorsed by physicians, the patients prefer a consultation. This, they say, is because they fear impersonal advice from the digital tool:

Digital tools are less personal. It's not about me it's about anyone. (P7)

The patients feel uncertain if the information in the digital tool can truly be transferable to their situation, as all patients and all cancer tumors are unique. They question how they can trust that the digital tool takes into consideration all the unique aspects of their situation in the way a human being would.

I want a human being to have the overall responsibility. As a patient I want to meet a person who can understand me in a way a digital tool never can. (P9)

According to the HCPs, there is a potential of putting strain on the patients by leaving them to themselves with all this information. They fear that the patients may not contact a nurse or physician because they believe they have figured things out themselves, resulting in misunderstanding or insufficiently informed patients.

There is no feedback in the chatbot. And they may think they have received all the information they needed, but perhaps they haven't. (HCP9)

The HCPs further highlight the importance of face‐to‐face contact. They emphasize that a chatbot cannot convey compassion or empathy, and that subtle signals patients express, signals they have learned to recognize through years of clinical experience, are easily missed by a digital tool. They all emphasize the importance of the human encounter.

I do believe that this particular contact between healthcare personnel and patients… that digital tools must not come at the expense of that. (HCP8)

This is further explained by another saying:

I don't think these digital solutions are as good as an in‐person consultation, precisely because they don't capture how the patients feel or what they are going through. (HCP5)

## 4 DISCUSSION

In this study, we interviewed both patients and HCPs about the use of the Rosa chatbot in a mainstream setting. Both groups spoke positively about the chatbot, describing it as user‐friendly, useful, available, safe, professional, and trustworthy. HCPs rated it higher than the Google search engine for providing genetic information in this context and recognized it as a valuable support tool. Although patients seek factual and thorough information, their primary concern is their cancer diagnosis. They feel overwhelmed and unable to absorb all the information at once. In this regard, they value the chatbot as a reliable and trusted support tool where they can access and revisit information at their own pace when they are ready. It enabled the patients to make informed decisions about preventing secondary cancers if a mutation was detected. However, one concern raised by the patients was the potential for misunderstandings when interacting with a digital tool. While such tools may offer highly accurate information, often endorsed by physicians, patients still prefer a personal consultation. They express a fear of receiving impersonal advice from the digital tool. They question how the tool can account for the unique aspects of their individual situations. Healthcare professionals (HCPs) share this fear, worried that the tool may not prompt clarifying questions and that it could lead to reduced patient contact.

During the interviews, many participants shared that they appreciated having access to genetic information through a chatbot for moments when questions arose. They expressed a strong need to remain in control of the information flow themselves, ensuring they would not receive more information than they felt able to process. Above all, they mainly wanted information that was relevant to their situation and offered a sense of hope. Many of the patients in our study expressed aversion towards searching the internet, afraid of being exposed to information they do not want, mentioning Rosa as a safe place to find information.

Mainstreaming genetic testing for cancer patients is considered feasible and beneficial both to patients and HCP (Berkman et al., 2025). The genetic test was something that the patients in our study were very grateful to be offered. However, they felt that extensive information about the test and implications of detecting a germline pathogenic variant was not relevant until after the test result was available and hereditary cancer had potentially been identified. The Rosa chatbot was mainly used for short questions in the MGT setting in our study; however, some had long and/or extensive conversations with Rosa, indicating it facilitates deeper engagement for those seeking more comprehensive information about MGT.

In a recent review (Lazarou et al., 2024) presenting patients' perspectives and requirements of digital health technologies, the authors highlighted the importance that the content presented through technologies was tailored and relevant to the patients' individual situation and was perceived as useful for the management of their own care. Digital tools can both support and challenge sound decision‐making (van Lingen et al., 2024). In‐person consultations following digital pre‐test counseling has been shown to enhance personalized decision‐making compared to in‐person counseling alone (Shickh et al., 2021). This resonates well with our findings of using Rosa in the MGT setting. Patients valued the chatbot for supporting their decision‐making in the event of a detected genetic mutation. They described learning what steps to take through the information provided by the chatbot.

The Rosa chatbot content focuses on aspects concerning hereditary breast and ovarian cancer and does not include content specifically tailored to the patient's cancer status. Although being informed about this, many participants asked questions related to treatment, prognosis, medications, side effects, and other topics beyond its current scope. This led to reduced chatbot performance, with higher levels of fallback responses and mismatched answers compared to previous evaluations (Siglen et al., 2023). This suggests that, to be more impactful, a chatbot designed for cancer patients could benefit from integrating diagnosis‐specific content alongside genetic information. This highlights a key consideration in designing digital information tools for affected individuals: while access to curated, high‐quality genetic information is valued, the perceived usefulness of a chatbot in a mainstream clinical setting may depend on broader content that extends beyond genetics. Balancing general hereditary cancer education with tailored information relevant to a patient's specific stage in their healthcare journey may enhance engagement and ensure that digital tools meet the diverse needs of users.

Notably there was no mention of technical issues using or accessing Rosa, a barrier raised previously in other patient‐centered chatbot studies (Fiallos et al., 2024). However, the fear of inaccuracies in the information provided by a chatbot was mentioned by some. This concern does not stem from Rosa providing incorrect information but rather from the perception that the information is generalized and not tailored to the individual's specific circumstances; thus, it is impersonal. Although conversational agents have demonstrated healthcare comparable to humans in other studies (Wutz et al., 2023), that was not confirmed in our study. Patients in clinical genetics value chatbots as a support tool for information that is difficult to find via regular internet searches (Luca et al., 2023), but it must not come at the expense of access to a clinician if they need individualized counseling or emotional support (Luca et al., 2023). Our patients felt overwhelmed by information and thus appreciated the chatbot as a reliable resource they could revisit at their own pace. The chatbot supported informed decision‐making regarding preventive measures after a potential positive genetic test. However, concerns were raised about the risk of misunderstandings and the potential impersonal nature of digital communication. HCPs also expressed apprehension that the chatbot might reduce opportunities for patient interaction and fail to prompt necessary clarifying questions. This fear represents a potential barrier to the implementation of chatbots in healthcare.

Our results suggest there is an emotional barrier to chatbot uptake; namely, the fear of missing out on human interaction. In human conversation, both parties can read between the lines and pay attention to facial expressions, as well as ask clarifying questions. In a conversation between patients and HCPs, the responsibility for the information provided is clearly allocated. Many participants in our study mentioned the need for a human to be responsible for what is said. This is supported by previous research (Lenz, 2021), showing that a digital tool cannot be held accountable as a human; thus, it should be allocated the role of an “assistant” (Lenz, 2021). HCPs also touch upon this in the interviews: How can we ensure that the relevant information is both understood and interpreted correctly? Even though the Rosa chatbot was introduced as a supplemental tool, the emotional barrier was evident. The participants expressed a certain skepticism about the ability of digital tools to provide sufficiently individualized information. While such tools can deliver general guidance, the participants questioned whether they could address the unique complexity of each cancer case, as a human clinician can. This perceived lack of personalization reinforces the preference for in‐person interactions. Healthcare professionals shared this concern, emphasizing that an overreliance on digital tools within the healthcare system could reduce direct patient contact. They cautioned that this might limit patients' opportunities to ask clarifying questions, potentially leading to misunderstandings or incomplete information.

This emotional barrier may interfere with successful implementation. Ensuring the chatbot serves as a supplement, allowing for face‐to‐face contact as needed, will therefore be central to successful implementation. Our study shows that a chatbot specific to the information need during MGT is relevant as the patients will have high return on investment, defined as the chatbot's “sweetspot” (Luca et al., 2023), and would enable patients to prepare themselves for an upcoming genetic counseling consultation if referred for one. As many clearly stated, if a pathogenic variant in one of the analyzed genes is detected, the chatbot will not suffice. This emotional barrier may lead to resistance towards chatbot use in healthcare. It is therefore essential to find strategies to manage this emotional barrier as we strive for hybrid health services.

Barriers to the implementation of information and communication technologies by HCPs in a clinical setting have been thoroughly investigated (Gagnon et al., 2012). Main barriers identified are: design or technical concerns, lack of compatibility with work processes, lack of familiarity with the information and communication technologies, time‐consuming or increased workload, and cost and legal issues (Gagnon et al., 2012), as well as considerations regarding data security and privacy (Mumtaz et al., 2023). Emotional barriers to health innovations applying to both patients and healthcare personnel include negative previous experiences, pre‐existing beliefs about effectiveness, lack of motivation, and resistance to change (Berardi et al., 2024). A qualitative systematic review of barriers and facilitators of mental healthcare technologies mentioned that providers may see technology as something interfering with the therapeutic alliance as well as causing job insecurity and concerns about overrelying on digital tools for decision‐making (Berardi et al., 2024). It has also been reported that HCPs fear that conversational agents will play such an important role in the future that they may replace humans, which in turn may reduce the quality of care (Wutz et al., 2023), as well as reduce job satisfaction as the physical meeting is also an important component of job engagement (Lee et al., 2024).

The fear of missing out on human interaction contributes to the otherwise documented barriers to the implementation of new technologies in healthcare. To overcome implementation barriers, participation of end‐users in the design, and the use of superusers during implementation to ensure proper training are suggested (Gagnon et al., 2012), as well as applying a problem‐centered approach, focusing on overcoming the practical implementation barriers (Mumtaz et al., 2023). The Rosa chatbot is developed with participatory design, involving end‐users in every step, and the user interface is evaluated as intuitive and user‐friendly (Siglen et al., 2022). Still, we identified an emotional barrier to implementation in the mainstream setting. The fear of missing out on human interaction may be explained if we look through the lenses of the Theory of Vulnerability (Boldt, 2019). In cases of severe disease, patients may experience elevated levels of cognitive vulnerability. Receiving a serious diagnosis can make it difficult to fully understand the medical information about the condition, including prognosis and treatment options (Boldt, 2019). It can be difficult to comprehend how these facts may affect daily routines, work, and other aspects of life (Boldt, 2019). HCPs are responsible for helping patients when they are ill. When they take the time to explain medical information clearly, using simple language and repeating information when necessary, they help reduce the patients' cognitive vulnerability (Boldt, 2019). The Rosa chatbot has the potential to alleviate symptoms of distress in the MGT setting by providing genetic information in simple language, allowing for reading and repeating as one wishes. However, in cases of cognitive vulnerability, the presence and reassurance of a human being cannot be compromised (Boldt, 2019).

We further argue that the focus on participatory design during development and problem‐solving during implementation will not suffice, as this doesn't provide a framework for overcoming the emotional barrier for end users in clinical practice. Patient‐centered care has previously been highlighted as a way forward in the implementation of digital healthcare (Berardi et al., 2024) and may serve as a helpful framework ensuring best practice when implementing digital tools in health care. There is no universal definition of patient‐centered care, but most attempts at explaining the concept integrate patients' preferences, values, and beliefs into the process of decision‐making, aiming at engaging patients in making informed and active choices (Grover et al., 2022). Patient‐centered care thus provides a framework that goes beyond applying participatory design by placing a heavy emphasis on the process of shared decision‐making regarding one's own health between the patient and the healthcare professionals. With patient‐centered care comes patient‐centered communication where active listening, the use of open‐ended questions, information‐sharing, and empathy are central components (Grover et al., 2022; Kvåle & Bondevik, 2008), relatable to genetic counseling. A focus on patient‐centered care may help HCPs maintain the highest level of expertise due to improved communication skills and enhanced decision‐making where the patients are informed and involved (Jaensch et al., 2019). Thus, a chatbot may serve as a facilitator of patient‐centered communication providing the amount of information the patient seeks at the time the patient wants, sharable with relatives and loved ones. This focus may in turn provide HCPs with continuous learning and a holistic understanding of the patient (Grover et al., 2022), thus potentially relieving the fear they have of missing out on patient feedback. Consequently, the patients will be reassured that the chatbot is a support tool serving to enhance care, not meant to compromise their access to qualified personnel. Well‐informed patients are better prepared to engage in discussions regarding their own health (Jaensch et al., 2019; Kane et al., 2015). The fear of missing out on human interaction may then diminish, and a genetic information chatbot may be embraced as a support tool enhancing care and fill a much‐needed information gap in MGT.

### 4.1 Strengths and Limitations

To our knowledge, this is the first published study presenting cancer patients' and their healthcare personnel's experiences with using a chatbot as a conversational tool during MGT. We have interviewed 17 patients and HCPs altogether using semi‐structured, yet open, interview guides, allowing for the participants' views and experiences to come forth. By using the SDI model in the data analysis, we ensure empirical coding and transparency at every step. With this approach, we aim to minimize the influence of the researchers' preconceived notions on the results as much as possible. Since three of the authors are either genetic counselors or geneticists, it has been important throughout the project to be aware of our preconceptions and implement strategies to manage them. This was central to the choice of using the SDI model.

All participants were recruited from the same geographical region in Norway. It would have strengthened the study if it had been expanded to include patients representing the entire country. This was argued against in the planning of this project, as we wanted to be sure we had the capacity to quickly take care of those patients experiencing stress or discomfort related to using the chatbot in a mainstream setting. We had no such inquiries. It may be argued that the sample size per group (patients vs. HCPs) is small; however, the two samples complement each other and together provide valuable insights to how a chatbot can best be implemented in the care of newly diagnosed cancer patients who are eligible for genetic testing. When reviewing the code list from the HCP transcripts, we found that no new themes emerged from the final three interviews; rather, they reinforced themes that had already begun to take shape. For the patient interviews, the saturation point was less clear. However, during the fifth step of the SDI model, when concepts were generated by analyzing both groups together, the crystallization of the core elements in the material became evident.

The patient sample of seven consisted of one known *BRCA* carrier. Only 2%–3% of the breast cancer population will prove to have a pathogenic *BRCA* variant (Grindedal et al., 2017; Høberg‐Vetti et al., 2016), making that subgroup quite rare. Furthermore, we were only able to recruit two ovarian cancer patients for the interviews. However, two interviews were excluded, one of which involved an ovarian cancer patient, because the participants had not used the chatbot. In general, ovarian cancer patients are older at the time of diagnosis than breast cancer patients and more often present with severe and advanced disease. The ovarian cancer group is underrepresented compared to breast cancer patients in our general sample for the overall study, and sadly some of them passed away shortly after their cancer diagnosis.

### 4.2 Implications for Practice and Future Research

This study shows that both patients and HCPs were positive towards using the Rosa chatbot in a mainstream setting. Both groups see benefits such as increased accessibility to information and enhanced efficiency but worry about possible drawbacks like reduced personal interaction and fear of misunderstandings. There is a strong emphasis on the need for face‐to‐face communication to provide empathy, validate concerns, and address unique patient needs. The participants emphasize the importance of balancing digital innovation with the preservation of essential human elements in healthcare delivery. This perspective contributes to the existing implementation models by highlighting an emotional barrier to adopting new technologies in healthcare. We further highlight patient‐centered care as a theoretical framework for successful implementation suitable to address and overcome barriers, also the emotional ones.

With this study, we hope to contribute to the ongoing discourse on the digitalization of healthcare. It emphasizes the importance of conducting thorough and critical discussions before transferring tasks traditionally performed by humans to digital platforms. A comprehensive understanding of the risks and benefits associated with such digital transitions is essential for all stakeholders. If the risks outweigh the benefits or are not fully understood, it may be advisable to refrain from digitizing these processes.

## 5 CONCLUSIONS

The Rosa chatbot is regarded as a valuable and trustworthy support tool by both cancer patients and healthcare professionals during MGT. Through the chatbot, they could choose what to read and when, thus also avoiding topics they did not want to read about just yet. Rosa was considered a valued support for decision‐making, providing accurate and timely information during the genetic testing process. However, patients also want human validation of the information, as they express not to fully trust a digital tool alone. The chatbot was described as impersonal, as it cannot provide genuine compassion and empathy. The patients worry about missing important information, receiving inaccurate guidance, or facing misunderstandings through digital platforms, and want responsibility for the information to remain with a healthcare professional. Healthcare professionals appreciate the benefits of digital tools but are concerned that patients may refrain from contacting a nurse or physician, believing they have understood everything on their own, potentially leading to misunderstandings or insufficiently informed decisions. Our findings contribute to the implementation model by identifying an emotional barrier described by both patients and HCPs. Addressing this barrier is central to ensure the successful implementation of the increasing number of digital tools in modern healthcare.

## AUTHOR CONTRIBUTIONS

Elen Siglen: Conceptualization, methodology, formal analysis, writing the original draft, review and editing. Hildegunn Høberg Vetti: Conceptualization, supervision, review and editing. Anita Lyssand: Writing, review and editing. Tone Dahl‐Michelsen: Conceptualization, supervision, methodology, writing, review and editing. Cathrine Bjorvatn: Conceptualization, supervision, funding acquisition, writing, review and editing. Tone Dahl‐Michelsen and Cathrine Bjorvatn contributed substantially and share last authorship. All Authors approved the final version of the manuscript.

## FUNDING INFORMATION

This study is funded by the Norwegian Cancer Society (grant number 194750) and is approved by the Regional Committee for Medical and Health Research Ethics in Western Norway (project number 2019/763).

## CONFLICT OF INTEREST STATEMENT

The authors declare to have no conflicts of interest.

## ETHICS STATEMENT

All participants in the study have signed an informed consent. The patients could also contact a genetic counselor available daily for any questions about the study, written materials, or chatbot content. All user communication with the platform is anonymous. It is not possible to match questions posed with the person posing.

## Data Availability

The data sets generated and analyzed during this study are not publicly available owing to privacy regulations but may be available from the corresponding author upon reasonable request.

## Footnotes

^1^
Cathrine Bjorvatn, Hildegunn Høberg Vetti and Tone Dahl‐Michelsen.
